# Measuring Misophonia: Assessing the Psychometric Properties of the MisoQuest and Its Ability to Predict Cognitive Impacts of Triggering Sounds

**DOI:** 10.1002/jclp.70033

**Published:** 2025-08-18

**Authors:** Kate E. Raymond, Blake E. Butler

**Affiliations:** ^1^ Graduate Program in Psychology University of Western Ontario London Ontario Canada; ^2^ Department of Psychology University of Western Ontario London Ontario Canada; ^3^ National Centre for Audiology University of Western Ontario London Ontario Canada; ^4^ Western Institute for Neuroscience University of Western Ontario London Ontario Canada

**Keywords:** cross‐cultural validation, misophonia, MisoQuest, reading comprehension, Stroop effect

## Abstract

**Objectives:**

Misophonia is characterized by an aversion to specific sounds, such as chewing and breathing. These “trigger” sounds can elicit negative emotional reactions, physiological stress, and cognitive impairments in people with misophonia. Despite its impact, misophonia lacks formal diagnostic classification, largely due to challenges in conceptualization and assessment. One of the few psychometrically robust self‐report measures for misophonia (the MisoQuest) was originally developed and evaluated in Polish. The current study evaluated the utility of the English language version of the MisoQuest, including assessment of its criterion validity using cognitive performance as an outcome.

**Methods:**

A total of 139 participants (44 people with misophonia and 95 controls) completed the MisoQuest, the Selective Sound Sensitivity Syndrome Scale (S‐Five), the Generalized Anxiety Disorders Scale, and the Sensory Hypersensitivity Scale. Participants then completed either a Stroop task or reading comprehension task in the presence/absence of triggering sounds. A subset of participants retook the MisoQuest after 5 weeks.

**Results:**

The MisoQuest showed excellent internal consistency and strong test‐retest reliability. Additionally, MisoQuest scores strongly correlated with S‐Five scores, supporting convergent validity, and moderately correlated with measures of generalized anxiety and sensory hypersensitivity, indicating some overlap while supporting discriminant validity. Higher MisoQuest scores predicted poorer reading comprehension performance when trigger sounds were present, supporting criterion validity. However, MisoQuest scores showed no significant relationship with Stroop task performance.

**Conclusion:**

These findings support the MisoQuest as a reliable and useful measure of misophonia in English‐speaking individuals and suggest its scores may relate to clinically relevant outcomes.

## 1 Introduction

Misophonia is a psychologically distressing condition involving an extreme aversion to certain ordinary sounds. The clinical presentation of misophonia was first described by Jastreboff and Jastreboff in 2001, and has since garnered increasing awareness among clinicians and researchers. Despite consensus among researchers that misophonia is a discrete disorder, there remains a lack of broader consensus regarding how misophonia should be characterized and assessed (Swedo et al. 2022).

### 1.1 Misophonia Characterization

Misophonia is a disorder characterized by an aversion to specific “trigger” sounds (Swedo et al. 2022). While trigger sounds vary between individuals with misophonia, they are commonly human‐produced, repetitive sounds like chewing and breathing (Claiborn et al. 2020; Rouw and Erfanian 2018). When someone with misophonia hears a trigger sound, it elicits a physiological stress response and feelings of intense anger, anxiety, and/or disgust (Edelstein et al. 2013; Kumar et al. 2017; A. Schröder et al. 2013).

Variation in emotional responses to sound is normal, but misophonic reactions can be functionally impairing and clinically significant. Although there are no agreed‐upon diagnostic criteria for misophonia, proposed symptoms include: aversive emotional and physiological reactions to certain sounds, loss of self‐control, insight into the excessive nature of these reactions, behavioural avoidance, and functional impairment (Jager et al. 2020). Some individuals affected by misophonia describe experiencing pain, suicidal ideation, and/or an inability to enjoy life because of their ongoing symptoms (Alekri and Al Saif 2019; Hocaoglu 2018). In addition, behavioral studies demonstrate that people with misophonia experience impaired attention and memory in the presence of trigger sounds when compared to healthy controls (Daniels et al. 2020; Frank et al. 2020; Seaborne and Fiorella 2018; Silva and Sanchez 2019). These trigger‐specific cognitive deficits represent functional impairments that are central to the conceptualization of misophonia. Accordingly, validated assessments for misophonia should produce scores that are meaningfully associated with these deficits.

Evidence‐based clinical assessments and interventions are imperative to the management and treatment of misophonia. However, misophonia is not currently listed in diagnostic manuals such as the Diagnostic and Statistical Manual for Mental Disorders (DSM; Gold 2014). As a result, individuals with misophonia are often misdiagnosed and left without evidence‐based treatment options (Brout et al. 2018; Claiborn et al. 2020; Potgieter et al. 2019). This is particularly concerning, as misophonia has been estimated to affect a staggering 5%–20% of individuals (Dixon et al. 2024; Jakubovski et al. 2022; Kılıç et al. 2021; Naylor et al. 2021; Vitoratou et al. 2023; Wu et al. 2014; Zhou et al. 2017). This point prevalence is alarmingly high given the potential severity of misophonia; however, these estimates were not derived from diagnostic criteria. Rather, the prevalence of misophonia has been estimated using self‐report measures that do not have well validated clinical cut‐offs (Dixon et al. 2024; Jakubovski et al. 2022; Kılıç et al. 2021; Naylor et al. 2021; Wu et al. 2014; Zhou et al. 2017). Thus, although 20% of the population *may* experience misophonia symptoms, prevalence estimates vary greatly between studies, and clinically significant cases are rarer (Dixon et al. 2024; Jakubovski et al. 2022). While there has been an influx of misophonia research in recent years (e.g., a 2023 collection in Frontiers in Neuroscience titled ‘Advances in Understanding the Nature and Features of Misophonia’ comprised 24 papers), the literature remains relatively limited, with fewer than 100 empirical articles published as of 2022 (Swedo et al. 2022). As a result, much remains unknown about the condition. Validated measures for misophonia are therefore crucial for accurately capturing prevalence, improving clinical practice, and advancing research in this developing field.

### 1.2 Misophonia Assessment

The most widely used self‐report measures for misophonia are the Amsterdam Misophonia Scale (A‐MISO‐S; Schröder et al. 2013) and the Misophonia Questionnaire (MQ; Wu et al. 2014). The development of these measures played a pioneering role in misophonia assessment, yet evidence supporting their validity remains limited. The A‐MISO‐S consists of six items that were adapted from the Yale‐Brown Obsessive‐Compulsive Scale (Goodman 1989) rather than being generated through psychometric analysis. While the MQ *was* developed through factor analysis, the MQ's Misophonia Severity Scale is a single item adapted from the National Institute of Mental Health Global Obsessive‐Compulsive Scale (Murphy et al. 1982). Since the development of these measures, the conceptualization of misophonia as an obsessive‐compulsive disorder (OCD) has been criticized. Research suggests that while misophonia is comorbid with OCD, it also co‐occurs with other psychological disorders, including mood disorders, anxiety disorders, post‐traumatic stress disorder, and attention deficit hyperactivity disorder (Claiborn et al. 2020; Jager et al. 2020; Rouw and Erfanian 2018). In addition, OCD symptoms do not appear to correlate with misophonia‐related impairment (Remmert et al. 2022). The generalizability of the A‐MISO‐S and MQ for use with the broader adult population is also uncertain, as validation studies have been conducted exclusively in youth, high school, and undergraduate samples (Cervin et al. 2023; Naylor et al. 2021; Sarigedik and Gulle 2021; Wu et al. 2014). Finally, to our knowledge, the test‐retest reliability of the A‐MISO‐S and MQ has yet to be established.

To address the absence of a well‐validated measure for misophonia, Siepsiak, Śliwerski, et al. (2020) developed the MisoQuest ‐ a self‐report measure originally written in Polish and validated in Polish‐speaking samples. The authors wrote 60 items that mapped onto the seven diagnostic criteria proposed by Schröder and colleagues (2013). They subsequently conducted Item Response Theory analysis to select items that effectively discriminated between those with and without misophonia (Siepsiak, Śliwerski, et al. 2020). The resulting MisoQuest is a unidimensional scale comprising 14 Likert‐rated items that tap a single underlying construct.

The MisoQuest was shown to have strong internal consistency (Cronbach's alpha = 0.95) and scores were highly stable over a period of 5 weeks (interclass correlation coefficient = 0.84; (Siepsiak, Śliwerski, et al. 2020). In addition, these psychometric data were acquired from an age‐diverse sample (18–68 years), providing generalizability for use with the broader adult population (Siepsiak, Śliwerski, et al. 2020). A receiver operating characteristic analysis of the data provided by Siepsiak, Śliwerski, et al. (2020) indicated that a cut‐off score of 61 resulted in the highest overall accuracy at discriminating controls (MisoQuest scores between 14 and 60) from people with misophonia (MisoQuest scores ≥ 61; Enzler et al. 2021). In a follow‐up study using that cut‐off value, nearly all participants who received a MisoQuest score of 61 or greater were diagnosed with misophonia using a structured clinical interview, indicating that the MisoQuest has high specificity (96.3%; Siepsiak, Sobczak, et al. 2020). However, a number of participants who were diagnosed with misophonia using the structured interview scored lower than 61 on the MisoQuest, indicating poorer sensitivity (66.67%; Siepsiak, Sobczak, et al. 2020).

In addition to the MisoQuest, several other self‐report measures have recently been developed to assess misophonia. This includes the Selective Sound Sensitivity Syndrome Scale (S‐Five; Vitoratou et al. 2021), the Duke Misophonia Questionnaire (DMQ; Rosenthal et al. 2021), and the Berlin Misophonia Questionnaire Revised (BMQ‐R; Remmert et al. 2022), which also have promising psychometric properties. The concurrent validation of multiple measures for misophonia represents substantial progress in misophonia assessment. Importantly, different measures assess misophonia at varying levels of specificity—some capturing the construct as a broad, higher‐order phenomenon, with others (such as the S‐Five) distinguishing between subcomponents like internalizing symptoms, externalizing symptoms, perceived threat and avoidance behaviours, outbursts, and impact on functioning. Having access to multiple validated measures is essential for researchers and clinicians, as different tools may be more suitable for certain settings, samples, and purposes. The MisoQuest assesses misophonia severity as a higher‐order construct and, with its validated cut‐off and concise item set, is particularly useful as a screener in research settings.

Overall, the MisoQuest appears to be a reliable and valid measure for identifying misophonia. However, the MisoQuest was written in Polish and validated in Polish‐speaking samples before being translated into English by the original research team. When a measure is translated into a different language, the translation process can introduce differences in meaning and cultural context; accordingly, it is crucial to re‐evaluate the reliability and validity of the translated measure (Brislin 1970). Some studies have since used the English language MisoQuest to assess misophonia severity without validating the measure (Enzler et al. 2021; Savard et al. 2022). Savard and colleagues (2022) reported the internal consistency of the MisoQuest (Cronbach's alpha = 0.89); however, a full cross‐cultural validation study has yet to be conducted and the properties of the English language MisoQuest remain unknown.

### 1.3 The Present Study

The goal of this study was to evaluate the utility of the English language version of the MisoQuest as a measure of misophonia by assessing its: (1) psychometric properties (factor structure, internal consistency, and test‐retest reliability); (2) convergent and discriminant validity; and (3) criterion validity through categorical (comparing cognitive performance between individuals classified with and without misophonia, based on the proposed clinical cut‐off) and dimensional approaches (testing whether MisoQuest scores predict cognitive performance deficits in the presence of trigger sounds, independent of general anxiety and sensory hypersensitivity).

To address aim three, the current study used two cognitive tasks: a Stroop task and a reading comprehension task. Both tasks have been used to identify trigger‐specific cognitive deficits in those with misophonia (Daniels et al. 2020; Seaborne and Fiorella 2018), but are considered to assess different cognitive processes. The Stroop task is used widely throughout psychology research as an indicator of cognitive control and selective attention (Bugg 2012), whereas reading comprehension is an indicator of sustained attention and memory‐related processes (Palladino et al. 2001). Using both tasks, the current study aimed to evaluate the type of cognitive deficits that are associated with MisoQuest scores.

In accordance with the psychometric data supporting the original version of the MisoQuest, we hypothesized that the English language MisoQuest would have a unidimensional factor structure with strong internal consistency and test‐retest reliability after 5 weeks. Next, we hypothesized that scores on the MisoQuest would be strongly correlated with scores on another misophonia measure (the S‐Five) but only weakly or moderately correlated with scores on measures for anxiety (the General Anxiety Disorder scale [GAD‐7]) and sensory hypersensitivity (the Sensory Hyperactivity Scale [SHS]). We hypothesized that individuals scoring 61 or greater on the MisoQuest (the previously identified cut‐off score for clinically‐relevant misophonia) would perform worse than control participants on the Stroop and reading comprehension tasks in the presence of trigger sounds. Finally, we hypothesized that MisoQuest scores would predict cognitive performance on the Stroop and reading comprehension tasks in the presence of trigger sounds across the spectrum of symptom severity, independent of scores on measures for anxiety and sensory hypersensitivity.

## 2 Methods

This study was approved by the University of Western Ontario's Non‐Medical Research Ethics Board. Study hypotheses and procedures were pre‐registered prospectively before data were collected on the Open Science Forum; see https://osf.io/nxhuk. Raw data, supporting materials, and task code are available at https://osf.io/3pf68/.

### 2.1 Participant Characteristics

To calculate the required sample size for this study, an *a priori* power analysis was conducted using G*Power (Faul et al. 2007). This analysis was designed to estimate the sample size required to perform a fixed model linear multiple regression with a power of 0.8 and a significance threshold of *α* = 0.05 for each cognitive task. The results suggested that a minimum sample size of 90 participants (45 per cognitive task) would be required to detect a medium effect size (Cohen 1988). We expected to observe a medium effect size because similar effects have been observed in previous laboratory studies of cognitive functioning in people with misophonia (Frank et al. 2020; Guetta et al. 2022; Simner et al. 2021).

**Figure 1:**
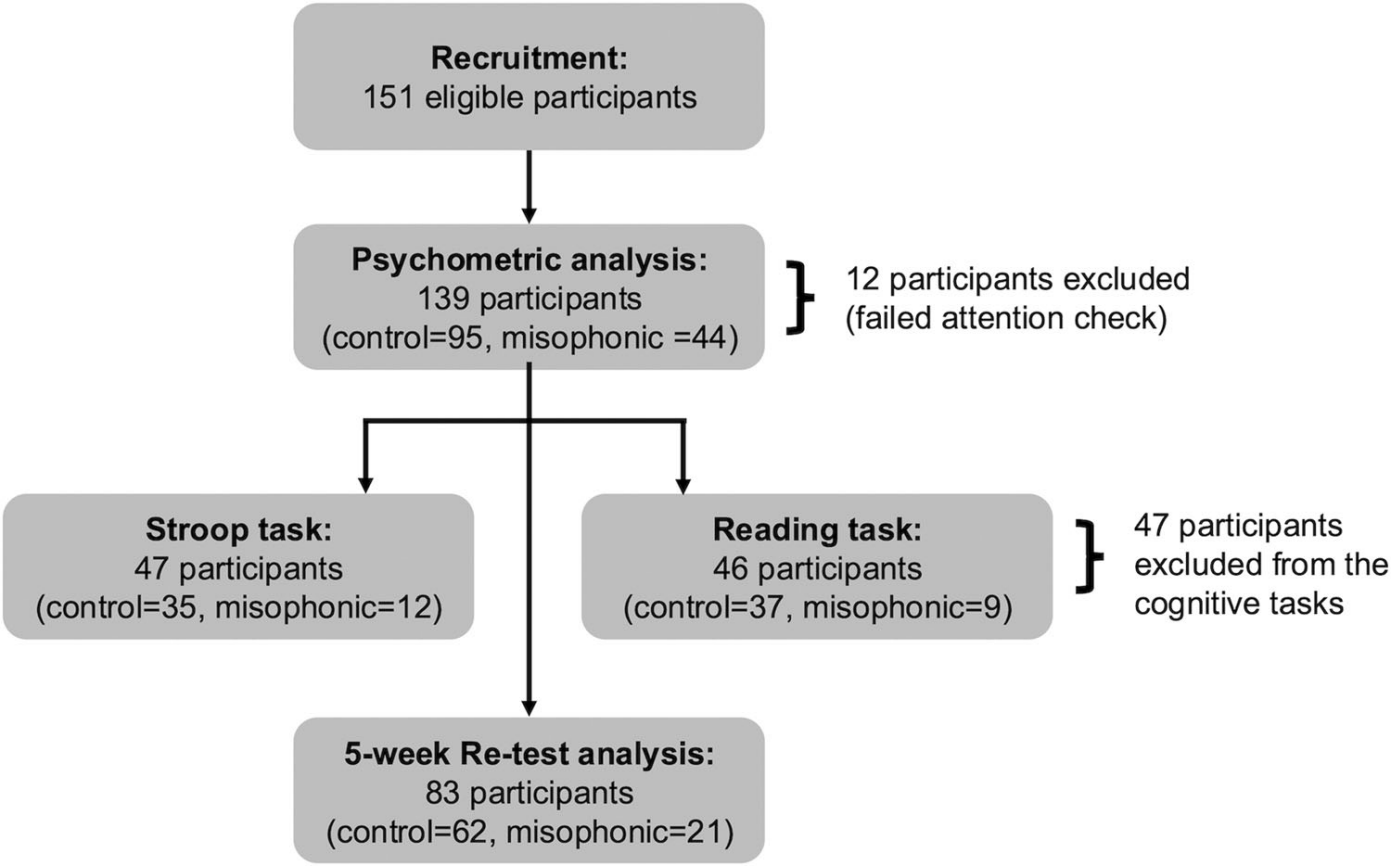
Participant flowchart.

**Figure 2:**
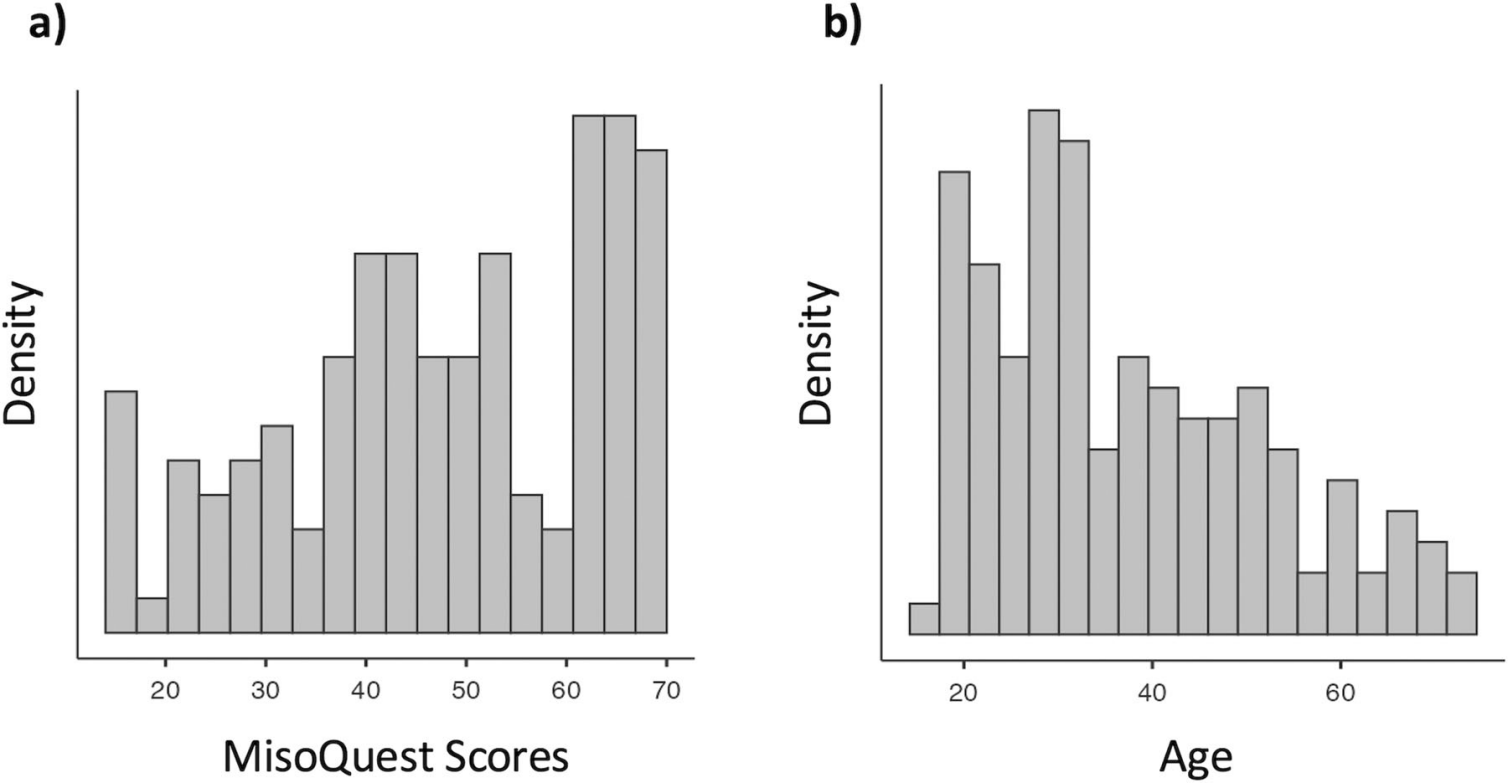
Distribution of sample. (a) MisoQuest scores and (b) age.

Participants were recruited through CloudResearch (New York, NY), the University of Western Ontario's SONA psychology research participation pool, and online advertisements circulated to misophonia support groups (including the SoQuiet misophonia research pool). Inclusion criteria required participants to be at least 17 years old, proficient in English, and have normal or corrected‐to‐normal vision. Participants were excluded if they reported a known hearing impairment. A total of 151 participants were recruited (Figure 1); however, after excluding participants who did not pass the attention checks (described below), a sample of 139 participants was included in the analyses. Forty‐four participants met the MisoQuest cut‐off for clinically significant misophonia and the remaining 95 participants comprised the control group (Table 1 and Figure 2). The sample was 65% White/Caucasian, 9% East Asian, 9% South Asian, 8% Black/African Descent, 5% Latinx/Hispanic, 3% Middle Eastern, and 1% Indigenous Peoples of Canada. Participants also reported their total household income over the past year: 17% of the sample reported an income less than $25,000, 20% reported $25,000–$49,999, 32% reported $50,000–$99,999, 27% reported $100,000–$199,999, and 4% of the sample reported an income greater than $200,000.

**Table 1:** Participant characteristics.

	Control (*n* = 95)	Misophonia (*n* = 44)
MisoQuest score	Range: 14–59 (mean = 39.5, SD = 12.3)	Range: 61–70 (mean = 65, SD = 2.96)
Age	Range: 18–73 (mean = 36.5, SD = 14.8)	Range: 17–72 (mean = 39.5, SD = 14.8)
Gender	63% female, 33% male, 2% transgender male, 2% non‐binary	83% female, 9% male, 2% non‐binary, 2% gender non‐conforming, 2% agender

### 2.2 Procedure

The study was conducted online using participants' personal laptop/desktop computers. Interested and eligible participants were provided a link to a survey hosted on Qualtrics (Provo, UT). Informed consent was collected, and participants were prompted to complete a self‐report assessment battery that included a demographics questionnaire as well as measures for misophonia severity (the MisoQuest and S‐Five), generalized anxiety (GAD‐7), and sensory hypersensitivity (SHS). Four attention checks were embedded throughout this battery that required participants to select a specific response option (e.g., Please select ‘7’) to indicate that they were reading and responding to each item. Participants who did not select the appropriate response on any of the four attention checks were excluded from all analyses (*n* = 12).

Once participants completed the assessment battery, they followed a link to one of two behavioral tasks (*either* a Stroop *or* reading comprehension task) written in PsychoPy/PsychoJS (Peirce et al. 2019) and hosted on Pavlovia (Nottingham, UK). Participants were assigned to only one task to reduce participant burden and minimize potential distress associated with exposure to trigger sounds. To minimize environmental variability, participants were asked to dim their lights, turn up the brightness on their computer screen, and minimize distractions. Participants were also encouraged to use headphones and to adjust their computer volume to a level that was loud but not uncomfortable (an example study sound was provided to help them calibrate their volume appropriately). Participants then completed three blocks of their assigned task, each under a different background sound condition: silence; a generally aversive sound (baby crying); or a common misophonic trigger sound (a person chewing gum). The three sound conditions were presented to participants in a random order and were counterbalanced across participants to control for order effects. After completing their assigned behavioral task, participants were asked if at any point during the experiment they had turned the sound off, or reduced the volume such that they could not hear the sounds. Participants who responded “yes” to this question, those who did not complete the task in its entirety, or those who reported encountering technical issues that would preclude the interpretation of their data were excluded from the behavioral analyses (*n* = 46). Finally, 5 weeks following the completion of the initial study, participants received a new link hosted on Qualtrics and were asked to complete the MisoQuest a second time.

### 2.3 Measures

#### 2.3.1 Demographics Questionnaire

This questionnaire collected demographic information about a participant's age, gender, ethnicity, education level, household income, and mental health diagnoses. This information was used to assess group equivalence and determine the generalizability of study results. An independent samples *t*‐test was conducted to compare age between the misophonic and control groups, and indicated no significant difference in age (*t*[137] = −1.11, *p* = 0.270). Chi‐square tests of independence were performed to examine relationships between group (misophonic vs. control) and categorical demographic variables. No significant associations were found for ethnicity (*χ* ^2^[13, *N* = 139] = 19.6, *p* = 0.106), education (*χ* ^2^[8, *N* = 139] = 8.59, *p* = 0.378), or mental health diagnoses (*χ* ^2^[50, *N* = 139] = 54, *p* = 0.324). However, the results indicated significant associations for gender (*χ* ^2^[5, *N* = 139] = 13.9, *p* = 0.016, Cramer's *V* = 0.316) and household income (*χ* ^2^[4, *N* = 139] = 12.1, *p* = 0.017, Cramer's *V* = 0.296). Visual inspection suggested that a larger portion of the misophonic sample identified as female and reported a higher household income than the control group. This partially aligns with previous research, which has found misophonia may be more prevalent in females (Dixon et al. 2024).

Education was used as a proxy for baseline cognitive ability in the regression models, where participants' education level was categorized as low (some primary, completed primary, some secondary, secondary degree), medium (some university but no degree, vocational degree), and high (bachelor's degree, graduate or professional degree). This categorical predictor was dummy‐coded, with low education as the reference category, creating two binary comparison variables: medium versus low and high versus low.

#### 2.3.2 MisoQuest

The MisoQuest (Siepsiak, Śliwerski, et al. 2020) is a 14‐item self‐report survey that assesses misophonia symptom severity, with questions about emotional reactions to sound and resulting distress and social impairment. The items are rated on a 5‐point Likert scale (from 1 = *I definitely do not agree* to 5 = *I definitely agree*) and are summed to produce a total score ranging from 14 (no misophonia symptoms) to 70 (severe misophonia symptoms). Scores greater than or equal to 61 indicate the presence of clinically significant misophonia (Enzler et al. 2021; Siepsiak, Śliwerski, et al. 2020). The MisoQuest was developed in Polish and translated into English by the original research team in collaboration with other researchers and interpreters.

#### 2.3.3 Selective Sound Sensitivity Syndrome Scale (S‐Five)

Participants' misophonia severity was also quantified using the Selective Sound Sensitivity Syndrome Scale (S‐Five; Vitoratou et al. 2021) to evaluate convergent validity. The S‐Five is a comprehensive, clinically‐oriented self‐report measure that assesses misophonia severity using 25 items distributed across five subscales: internalizing, externalizing, perceived threat, outbursts, and impact. The items are rated on an 11‐point scale (from 0 = *not at all true* to 10 = *completely true*) and are summed with higher overall scores indicating greater misophonia severity. The S‐Five has strong internal consistency, test‐retest reliability, and preliminary evidence of convergent and discriminant validity (Vitoratou et al. 2021, 2023).

The S‐Five‐T trigger checklist was used to assess the type (i.e., no feeling, irritation, distress, disgust, anger, panic, other negative feeling, or other positive feeling) and intensity of participant responses to certain sounds. The intensity of response was rated on an 11‐point scale (from 0 = *doesn't bother me at all* to 10 = *unbearable/causes suffering*). A sound was considered a trigger if a participant reported reacting negatively to the sound in a manner beyond irritation (i.e., distress, anger, disgust, panic) and if they rated the intensity of this reaction as a 5 or greater.

#### 2.3.4 Generalized Anxiety Disorders Scale (GAD‐7)

The Generalized Anxiety Disorders Scale (GAD‐7; Spitzer et al. 2006) is a 7‐item unidimensional scale measuring worry and anxiety symptoms. The items are rated on a 4‐point scale (from 0 = *not at all* to 3 = *nearly everyday*) and are summed to produce a total score ranging from 0 (*no or minimal anxiety*) to 21 (*severe anxiety*). The GAD‐7 has excellent internal consistency and strong evidence of construct validity (Johnson et al. 2019).

#### 2.3.5 Sensory Hypersensitivity Scale (SHS)

The SHS (Dixon et al. 2016) is a 25‐item self‐report survey that assesses sensory over‐responsivity. The items are rated on a 5‐point Likert scale (from 1 = *strongly disagree* to 5 = *strongly agree*) and are summed with higher total scores indicating greater sensory over‐responsivity. The SHS assesses sensitivity across nine subscales, which correspond to different sensory stimuli, including light, sound, touch, taste, smell, pain, and temperature. The SHS has good internal consistency and evidence of discriminant validity (SHS scores weakly correlate with measures of depression and anxiety [Dixon et al. 2016]).

### 2.4 Behavioral Tasks and Materials

#### 2.4.1 Auditory Stimuli

For each sound category, a single stimulus was selected as an indicator rather than using an exhaustive set of sounds. The sound of an individual chewing gum was selected for the misophonic trigger condition because it includes several elements of triggering mouth sounds (e.g., chewing, lip smacking, and wet saliva sounds), which are among the most common triggers for misophonia (Enzler et al. 2021; Vitoratou et al. 2021). Indeed, one study found that 96.5% of individuals with self‐reported misophonia endorsed mouth sounds as one of their triggers (Claiborn et al. 2020). Similarly, the sound of a baby crying sound was selected for the aversive sound condition because it is universally considered to be unpleasant (Green et al. 1995) and is considered similarly aversive by individuals with and without misophonia (Kumar et al. 2017). The gum chewing sound file was 5 min and 10 s long and the baby crying sound file was 6 min and 56 s long. Neither sound file was looped, as their lengths exceeded the duration of the task blocks. Both sound files were retrieved from online sources and normalized to have the same root‐mean‐square amplitude. The stimuli have been made available on the Open Science Framework for reproducibility.

#### 2.4.2 Stroop Task

In each trial of the Stroop task, participants were presented with a color word (“red”, “green”, or “blue”) on their computer screen and instructed to use their keyboard arrow keys to indicate the color of the text as quickly and accurately as possible while ignoring the semantic meaning of the word (the mapping of colors to arrow keys was presented at the bottom of the screen as a reminder throughout the experiment). On congruent trials, the color of the text matched the meaning of the word (e.g., the word “red” written in the red text), while on incongruent trials, the color of the text differed from the meaning of the word (e.g., the word “red” written in blue text). Participants completed one block of 30 practice trials, followed by three blocks of 72 experimental trials. Each block was presented in one of the three sound conditions, where the sound played uninterrupted over the duration of the block. Within each experimental block, participants were presented with 36 congruent trials and 36 incongruent trials (in random order) with a 500 ms interval between trials. Participants were given the opportunity to take a break between experimental blocks and no sound was presented during the break.

The Stroop effect was quantified as the difference between a participant's mean reaction time on congruent trials and incongruent trials. Only trials where the participant responded correctly were included in this analysis. The magnitude of the Stroop effect for each sound condition was used as an index of cognitive control and selective attention, where a larger Stroop effect indicated poorer cognitive control and selective attention. Mean percent accuracy across all trials was also analyzed for each sound condition to supplement the interpretation of Stroop effect results.

#### 2.4.3 Reading Comprehension Task

In the reading comprehension task, participants read three short stories on their computer screen, each presented in one of the three sound conditions, with the sound played uninterrupted for the duration of the story. The three stories were “*The Pea Blossom*”, “*Sunshine Stories*”, and “*The Snowman*”—short children's fairy tales written by Hans Christian Andersen with similar word counts. The text of each story was presented across multiple screens, and participants progressed from one screen to the next by pressing the spacebar on their keyboard. Before beginning the experiment, participants were informed that they would be directed to answer a series of recall questions when they had completed each story, or after 5 min had elapsed (whichever came first). Following each story, participants were given as much time as they needed to answer 10 multiple choice questions (each with five response options: one correct and four incorrect). No sound was presented while participants were answering questions, the questions were presented on the computer screen one at a time, and participants were instructed to use their keyboard keys to select the response option they thought was correct. Participants were given the opportunity to take a break between experimental blocks and no sound was presented during the break.

Reading comprehension accuracy was quantified as the number of correctly answered multiple‐choice questions (out of 10). Reading comprehension accuracy for each sound condition was used as an indicator of sustained attention and working memory, where decreased accuracy indicated poorer sustained attention, and encoding.

### 2.5 Data Analytic Strategy

#### 2.5.1 Psychometric Analyses

The 14 items of the Polish MisoQuest load onto a single factor, meaning they assess a single underlying construct: misophonia severity (Siepsiak, Śliwerski, et al. 2020). To assess whether the factor structure of the English language MisoQuest is consistent with the Polish MisoQuest, confirmatory factor analysis (CFA) was conducted using a unidimensional model. The goodness‐of‐fit of this model to the data was evaluated using a Comparative Fit Index (CFI), Tucker‐Lewis Index (TLI), and Root Mean Square Error of Approximation (RMSEA). Internal consistency was evaluated using Cronbach's alpha to further assess the homogeneity of the MisoQuest.

To evaluate the convergent and discriminant validity of the MisoQuest, Pearson correlation coefficients were computed between each participant's MisoQuest score and their scores on the S‐Five, GAD‐7, and SHS. Finally, test‐retest reliability was evaluated by re‐administering the MisoQuest to participants 5 weeks following completion of the initial study and computing the interclass correlation coefficient.

#### 2.5.2 Behavioral Task Analyses

For categorical analyses, participants were divided into groups comprising those with and without clinically significant misophonia using the proposed cut‐off score for the MisoQuest (Siepsiak, Sobczak, et al. 2020). Participants with scores from 14 to 60 comprised the control group and participants with scores 61–70 comprised the misophonia group. A two‐way ANOVA with group (control, misophonia) as a between‐subjects factor and sound type (silence, aversive, trigger) as a within‐subjects factor was conducted for each task. The magnitude of the Stroop effect and reading comprehension accuracy were used as dependent variables in the two analyses. The standard alpha of 0.05 was used to determine the presence of a significant main effect or interaction, and type III sums of squares were specified to account for differences in sample sizes across groups. Since directional predictions were established a priori, we performed one‐tailed *t*‐tests to interpret significant effects where applicable.

In addition to the categorical analyses, we evaluated whether MisoQuest scores predicted task performance across the full range of sound sensitivity using multiple linear regression to control for general anxiety, sensory hypersensitivity, and education level. This analysis investigated the influence of four predictor variables (MisoQuest, GAD‐7, SHS, and education level) on each dependent variable (Stroop effect and reading comprehension accuracy in the presence of trigger sounds) using an alpha of 0.05 to assess the significance of regression models and predictors. In addition to the reported regression coefficients, Pearson correlations were also computed to evaluate the relationship between MisoQuest scores and performance outcomes. Since Pearson correlations are susceptible to outlier influence, bivariate outliers were identified by calculating the Mahalanobis distance for each observation in the correlation, which was compared to the critical value on the chi‐squared distribution using an alpha of 0.05. All correlation analyses were conducted before and after the removal of identified outliers and interpreted accordingly.

For each behavioural task, we tested two pre‐registered hypotheses: (1) that the misophonia group would have poorer cognitive performance than the control group, specifically in the presence of trigger sounds; and (2) that higher MisoQuest scores would predict lower cognitive performance, specifically in the presence of trigger sounds. Given that our analyses collectively evaluate these hypotheses about trigger‐specific deficits, rather than multiple independent hypotheses, we report unadjusted *p*‐values and include effect sizes for further interpretation.

## 3 Results

### 3.1 MisoQuest Psychometric Properties

A CFA was conducted to verify the factor structure of the English language MisoQuest. The standardized factor loadings, representing the correlation between each item and the underlying latent factor, ranged from 0.714 to 0.895 (Table 2). CFA using a unidimensional model yielded the following model fit indices: CFI = 0.920, TLI = 0.906, and RMSEA = 0.117. The CFI and TLI values suggest that the proposed unidimensional model is a good fit for the observed data (Hu and Bentler 1999). However, a RMSEA value of 0.117 suggests a poor fit, and indicates that the unidimensional model may not accurately reproduce the observed pattern of covariance (Hu and Bentler 1999). Given the disagreement between model fit indices, an exploratory factor analysis (EFA) was conducted using principal component analysis to determine if an alternative model may better fit the data. The resulting eigenvalues were as follows: 9.835 for the first factor, 0.770 for the second factor, 0.582 for the third factor, and so on, up to 0.091 for the 14th factor. The first factor alone accounted for 69.3% of the total variance, indicating that a unidimensional model is the best fit for our data. Finally, the internal consistency of the MisoQuest was high (Cronbach's alpha = 0.966), indicating that the items are strongly correlated with one another and reliably assess a common underlying construct.

**Table 2:** MisoQuest factor loadings.

Factor	Indicator	Factor loading
Misophonia severity	Item 1: Some sounds bother me so much that I have difficulty controlling my emotions.	0.895
	Item 2: Unpleasant sounds make me feel overwhelmed.	0.870
	Item 3: I become anxious at the mere thought of an unpleasant sound.	0.786
	Item 4: I believe that my reactions to sounds are exaggerated, but I can't get rid of them.	0.868
	Item 5: When I hear unpleasant sounds, I start sensing emotions in my body (e.g., I sweat, feel pain, feel pressure, my muscles tense).	0.840
	Item 6: I start feeling anger the moment I see a thing/animal/person that might make an unpleasant sound at any time.	0.714
	Item 7: I put a lot of effort into controlling emotions when I hear an unpleasant sound.	0.780
	Item 8: If I can, I avoid meeting with certain people because of the sounds they make.	0.770
	Item 9: I find some sounds made by the human body unbearable.	0.777
	Item 10: I feel that my mental state worsens if I cannot leave a place where there's an unpleasant sound.	0.864
	Item 11: I often think about how to drown out unpleasant sounds.	0.745
	Item 12: Some unpleasant sounds make me instantly angry.	0.864
	Item 13: I am scared that unpleasant sounds may impact my future.	0.762
	Item 14: When meeting with other people, I am sometimes irritated because of unpleasant sounds that are present.	0.834

**Figure 3:**
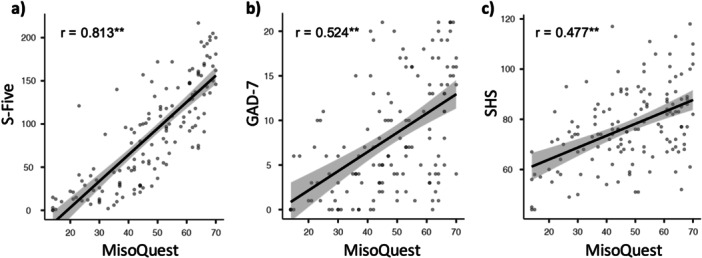
Pearson correlations between (a) MisoQuest scores and S‐Five scores; (b) MisoQuest scores and GAD‐7 scores; (c) MisoQuest scores and SHS scores. The shaded area represents the standard error, **p < 0.001.

The convergent validity analysis (Figure 3a) revealed a strong positive correlation between scores on the MisoQuest and S‐Five scores (*r* = 0.813, *p* < 0.001), indicating a high degree of convergence between measures designed to assess the same construct (misophonia severity). There were also significant positive correlations between MisoQuest scores and scores on the GAD‐7 (*r* = 0.522, *p* < 0.001; Figure 3b) and SHS (*r* = 0.476, *p* < 0.001; Figure 3c), indicating that individuals with more severe misophonia symptoms tend to have more severe anxiety and sensory hypersensitivity. Although these variables are meaningfully associated, the relations between scores on the MisoQuest and measures for distinct constructs (GAD‐7 and SHS) are weaker than the relation between scores on the MisoQuest and another measure for misophonia (S‐Five), thereby demonstrating discriminant validity.

**Figure 4:**
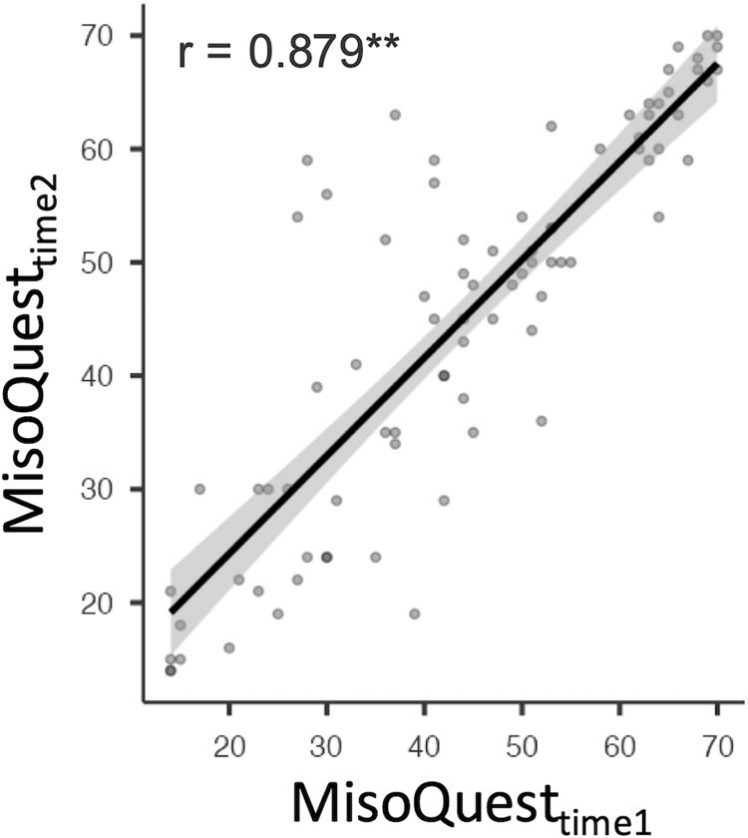
Pearson correlation between MisoQuest scores at timepoint 1 and MisoQuest scores at timepoint 2 (5 weeks later). The shaded area represents the standard error, **p < 0.001.

Finally, the test‐retest reliability analysis demonstrated consistent results over a 5‐week interval. The interclass correlation coefficient between MisoQuest scores obtained at the two timepoints was strong (ICC [68.0] = 0.88, *p* < 0.001, 95% CI = 0.82–0.92), indicating excellent agreement between the two timepoints. For illustrative purposes, a scatterplot showing the linear relationship between the scores at the two timepoints is presented (Figure 4), along with the Pearson correlation coefficient (*r* = 0.879, *p* < 0.001).

### 3.2 Behavioral Task Results

Of the 139 participants that completed the assessment battery, 46 were excluded from the behavioral analyses because they did not complete their assigned task in its entirety (14 controls, 13 people with misophonia), reported experiencing technical difficulties (2 controls), or reported turning their computer volume way down or off, such that they were not listening to the study sounds while completing the task (7 controls, 10 people with misophonia). In total, 47 participants were included in the Stroop task analyses (35 controls, 12 people with misophonia) and 46 participants were included in the reading task analyses (37 controls, 9 people with misophonia). An exploratory one‐way ANOVA was conducted to determine if the group excluded from behavioural analyses had differing misophonia severity from the groups included in the Stroop and reading comprehension tasks. The ANOVA revealed a significant main effect (*F*_[2, 90.5]_ = 5.85, *p* = 0.004), where the excluded group had significantly greater MisoQuest scores than the Stroop task group (*t*[91] = 8.89, *p*_tukey_ = 0.015) and the reading comprehension group (*t*[90] = 9.93, *p*_tukey_ = 0.006). This suggests that individuals with greater misophonia severity were disproportionately excluded from the behavioural analyses.

Notably, 88.6% of participants who met the MisoQuest cut‐off for misophonia endorsed gum chewing as a trigger, based on the S‐Five‐T (see the S‐Five section in methods for details). In the behavioural analyses, 91.7% of the misophonic sample who completed the Stroop task and 88.9% of the misophonic sample who completed the reading comprehension task endorsed gum chewing as a trigger. The remaining portion of the misophonic sample who completed each behavioural task found gum chewing irritating but at a reduced intensity. Overall, this demonstrates that the stimulus used for the misophonic trigger condition was likely to effectively elicit the expected response in most of the misophonic sample.

On average, it took participants assigned to the Stroop task 90.0 s to complete each experimental block and participants assigned to the reading comprehension task 227.6 s to complete each experimental block. This indicates that on average, participants in the reading comprehension task were exposed to each sound type for 2.5 times longer than participants in the Stroop task. These averages do not include breaks, the time it took participants assigned to the Stroop task to complete practice trials, or the time it took participants assigned to the reading comprehension task to answer multiple choice questions, all of which were completed in silence.

### 3.3 Stroop Task

**Figure 5:**
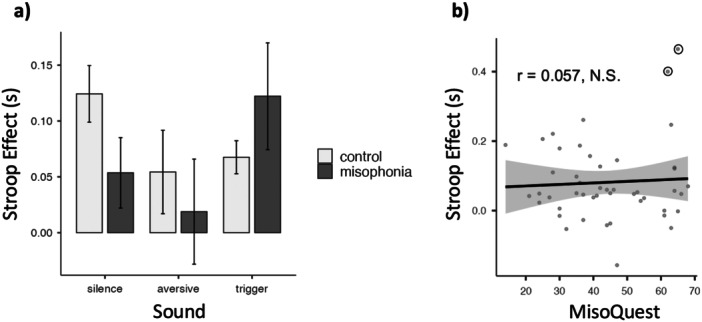
(a) Mean Stroop effect scores for the control group (light grey) and misophonia group (dark grey) for each sound condition. Error bars represent the standard error of the mean. (b) Pearson correlation between MisoQuest scores and Stroop effect magnitudes for the trigger sound condition. The shaded area represents the standard error; bivariate outliers are circled.

A repeated‐measures ANOVA conducted on Stroop effect magnitudes (the difference in reaction times between incongruent and congruent trials) revealed no significant main effect of group (*F*_[1, 45]_ = 0.192, *p* = 0.663, *η*² = 0.002), no significant main effect of sound type (*F*_[2, 45]_ = 2.13, *p* = 0.125, *η*² = 0.021), and no significant interaction (*F*_[2, 45]_ = 2.16, *p* = 0.121, *η*² = 0.021; Figure 5a). A repeated‐measures ANOVA conducted on overall accuracy values (the percentage of correct responses across all trial types) revealed no significant main effect of group (*F*_[1, 45]_ = 0.015, *p* = 0.903, *η*² = 0.000), no significant main effect of sound type (*F*_[2, 45]_ = 1.37, *p* = 0.259, *η*² = 0.001), and no significant interaction (*F*_[2, 45]_ = 2.29, *p* = 0.107, *η*² = 0.001).

To analyze the influence of four predictor variables (MisoQuest scores, GAD‐7 scores, SHS scores, and education level [low vs. medium and low vs. high]) on the outcome of interest (Stroop effect in the presence of trigger sounds), a multiple linear regression was conducted to determine the portion of variance in the outcome variable attributed to the combination of predictors. The overall regression was non‐significant (*R* = 0.203, *R* ^2^ = 0.0413, adjusted *R* ^2^ = −0.0816, *F*_[5, 40]_ = 0.336, *p* = 0.888). Furthermore, the Pearson correlation between MisoQuest scores and Stroop effect in the trigger condition was also non‐significant (*r* = 0.057, *p* = 0.706; Figure 5b). Two bivariate outliers were identified and subsequently removed from the analysis; however, the resulting correlation remained non‐significant (*r* = −0.186, *p* = 0.232).

#### 3.3.1 Reading Comprehension Task

**Figure 6:**
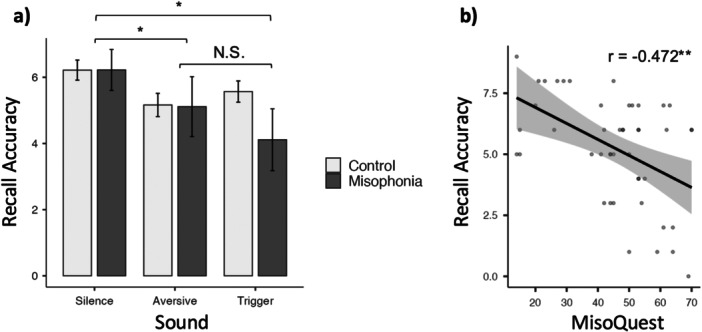
(a) Mean recall accuracies for the control group (light grey) and misophonia group (dark grey) for each sound condition. Error bars represent the standard error of the mean; *p < 0.05. (b) Pearson correlation between MisoQuest scores and recall accuracy for the trigger condition. The shaded area represents the standard error; **p < 0.001.

A repeated‐measures ANOVA conducted on reading accuracy scores revealed no significant main effect of group (*F*_[1, 44]_ = 0.696, *p* = 0.409, *η*² = 0.009), a significant main effect of sound type (*F*_[2, 44]_ = 5.85, *p* = 0.004, *η*² = 0.049), and no significant interaction (*F*_[2, 44]_ = 4.97, *p* = 0.155, *η*² = 0.016; Figure 6a). Thus, while there are no apparent group differences in reading accuracy scores between controls and people with misophonia, both groups performed worse in the presence of aversive (*t*[44] = 2.480, *p*_tukey_ = 0.044) and trigger sounds (*t*[44] = 3.035, *p*_tukey_ = 0.011) when compared to silence. To ensure accuracy scores were not confounded by a group difference in time‐on‐task (i.e., to determine whether people with misophonia were advancing through the screens of text more quickly than control participants in an effort to bring about the end of the trial more quickly), an exploratory analysis of reading time was performed. A repeated measures ANOVA revealed no significant main effect of group (*F*_[1, 44]_ = 0.000, *p* = 0.976, *η*² = 0.000), a significant main effect of sound type (*F*_[2, 44]_ = 5.44, *p* = 0.006, *η*² = 0.029), and no significant interaction (*F*_[2, 44]_ = 2.39, *p* = 0.098, *η*² = 0.013). Both groups spent more time reading in silence compared to aversive (*t*[44] = 2.757, *p*_tukey_ = 0.023) and trigger sounds (*t*[44] = 2.704, *p*_tukey_ = 0.026).

To analyze the influence of the four predictor variables (MisoQuest scores, GAD‐7 scores, SHS scores, and education level [low vs. medium and low vs. high]) on the outcome of interest (reading comprehension in the presence of trigger sounds) a multiple linear regression was conducted to determine the portion of variance in the outcome variable attributed to the combination of predictors. The overall regression was significant (*R* = 0.511 *R* ^2^ = 0.261, adjusted *R* ^2^ = 0.169, *F*_[5, 40]_ = 2.83, *p* = 0.028). To assess the contributions of individual predictors, the magnitude and statistical significance of the standardized regression coefficients were examined. MisoQuest scores significantly predicted the outcome of interest (*B* = −0.368, *p* = 0.038), while GAD‐7 scores (*B* = −0.049, *p* = 0.772), SHS scores (*B* = −0.143, *p* = 0.366), and education level (*B* = −0.525 to −0.627, *p* = 0.273–0.380) had non‐significant regression coefficients. This suggests that the severity of an individual's misophonia predicts reading comprehension accuracy in the presence of trigger sounds, independent from their GAD‐7 score, SHS score, and education level. The Pearson correlation between MisoQuest scores and reading comprehension accuracy scores was significant (*r* = −0.472, *p* < 0.001; Figure 6b), indicating that individuals with greater misophonia severity had lower reading comprehension accuracy in the presence of trigger sounds.

In contrast, the overall regression was non‐significant when reading comprehension in the presence of aversive sounds (*R* = 0.371, *R* ^2^ = 0.138, adjusted *R* ^2^ = 0.030, *F*_[5.0, 40.0]_ = 1.28, *p* = 0.292) and reading comprehension in silence (*R* = 0.276, *R* ^2^ = 0.076, adjusted *R* ^2^ = −0.040, *F*_[5, 40]_ = 0.658, *p* = 0.657) were used as outcome variables. This indicates that MisoQuest scores are not predictive of reading comprehension accuracy in non‐trigger sound conditions.

Given that cognitive performance may decline with age (Murman 2015), an exploratory multiple linear regression analysis was conducted with age as a predictor. To keep the number of predictors consistent across models, age replaced education level as the previously used indicator of cognitive ability. The overall regression was significant (*R* = 0.495, *R* ^2^ = 0.245, adjusted *R* ^2^ = 0.170, *F*_[5, 40]_ = 3.25, *p* = 0.021). MisoQuest scores significantly predicted the outcome of interest (*B* = −0.396, *p* = 0.025), while GAD‐7 scores (*B* = −0.014, *p* = 0.937), SHS scores (*B* = −0.147, *p* = 0.368), and age (*B* = −0.078, *p* = 0.586) had non‐significant regression coefficients.

## 4 Discussion

The objective of the current study was to advance misophonia assessment by conducting a cross‐cultural validation of the MisoQuest—a promising self‐report measure for misophonia. The study contributes to a growing body of evidence supporting the reliability and validity of the MisoQuest. It also provides novel insight into the criterion validity of the MisQuest by examining the relation between MisoQuest scores and cognitive impairments previously linked to misophonia, though findings were mixed. The MisoQuest was originally developed in Polish and had previously only been evaluated in Polish‐speaking samples; thus, the current study bridged a critical gap in misophonia assessment by evaluating the psychometric properties of the English language MisoQuest in an English‐speaking sample.

In alignment with the findings of Siepsiak, Śliwerski, et al. (2020), we demonstrated that the MisoQuest has excellent internal consistency and strong stability across a 5‐week interval. This indicates that the MisoQuest is a reliable measure for assessing misophonia symptom severity in English‐speaking individuals. In addition to replicating the analyses of Siepsiak, Śliwerski, et al. (2020), we sought to assess the convergent and discriminant validity of the MisoQuest. We demonstrated that MisoQuest scores are strongly correlated with scores on another measure of misophonia severity and only moderately correlated with scores on measures of anxiety and sensory hypersensitivity. Given that misophonia severity is known to be associated with the severity of anxiety (Bagrowska et al. 2022; Schadegg et al. 2021) and sensory hypersensitivity symptoms (Wu et al. 2014), moderate correlations between these measures likely reflect shared variance due to comorbidity between constructs. Thus, our results align with our hypotheses, provide preliminary evidence of convergent and discriminant validity for the MisoQuest, and suggest that the MisoQuest captures the misophonia construct beyond what is accounted for by anxiety or sensory hypersensitivity. This represents a crucial step towards ascertaining the overall construct validity of the MisoQuest. Overall, the psychometric evidence presented here supports the reliability and validity of the English language MisoQuest as a tool for assessing misophonia severity. These findings provide researchers and clinicians improved confidence in their ability to accurately assess misophonia severity in English‐speaking individuals, and will strengthen the claims of previous and future research studies using the MisoQuest. However, measurement validation is an ongoing process and researchers should continue to evaluate the factor structure, reliability, and validity of the MisoQuest in diverse samples.

The sample size for the current study was estimated a priori to ensure a medium effect could be detected in the regression analyses that examined the relationship between cognitive impairments and the full range of MisoQuest scores. However, the resulting samples were ultimately underpowered to detect group differences in cognitive outcomes on the Stroop and reading comprehension tasks due to the small number of individuals who reached the MisoQuest cut‐off score (*n* = 12 and *n* = 9 respectively, where *n* = 18 was deemed necessary to effectively power the analyses). The MisoQuest cut‐off score used to divide participants with and without clinically significant misophonia yields high diagnostic accuracy (Enzler et al. 2021) but sacrifices sensitivity (Siepsiak, Sobczak, et al. 2020), meaning that some individuals with misophonia were likely not captured using this cut‐off. The categorical approach to misophonia assessment may, therefore, make it challenging for researchers to recruit large enough misophonia samples to power their desired analyses.

When misophonia severity was treated as a dimensional variable, we found a graded association between MisoQuest scores and reading comprehension accuracy, such that higher scores were associated with lower accuracy in the presence of trigger sounds. Since trigger sounds were played during the encoding phase (when participants were reading the story), this result suggests that MisoQuest scores are associated with trigger‐specific deficits in sustained attention and working memory. Furthermore, MisoQuest scores were found to predict reading comprehension accuracy independent from general anxiety, sensory hypersensitivity, and education level. This finding strengthens the discriminant validity discussed earlier and provides evidence of criterion validity for the MisoQuest, by demonstrating that scores on this measure are related to clinically meaningful outcomes.

Contrary to previous findings, misophonia severity did not predict an increased Stroop effect when the trigger sound condition was considered in isolation (Daniels et al. 2020). This discrepancy may stem from measurement differences; Daniels and colleagues (2020) found that scores on the MQ Emotional Behaviours scale predicted Stroop effect magnitude, while the MisoQuest may not capture selective attention impairments. However, this discrepancy may also stem from broader methodological differences. For instance, the current study was conducted online, whereas Daniels and colleagues (2020) conducted their study in a controlled laboratory environment. Additionally, Daniels and colleagues (2020) used multiple sequentially played sounds, whereas we presented one continuously played sound for each sound type. This reduction in stimulus variation could have increased predictability and reduced anticipatory anxiety—a form of distress related to the anticipation of hearing a trigger sound that may contribute to the severity of misophonic symptoms (Dozier and Mitchell 2023).

While prolonged exposure to trigger sounds may theoretically lead to habituation, research suggests that prolonged exposure to trigger sounds may result in increased distress in individuals with misophonia (Ferrer‐Torres and Giménez‐Llort 2021; Frank and McKay 2019; Schröder et al. 2017). Notably, in the reading comprehension task (where significant effects emerged) participants were exposed to gum chewing sounds for 2.5 times longer on average than participants in the Stroop task. Thus, differences in observed effects between cognitive task types may be partially attributed to sound duration. It is also possible that trigger‐specific cognitive deficits may be more readily observed in tasks that require greater cognitive resources; indeed, the ability to ‘tune out’ irrelevant stimuli decreases with increased cognitive load (Sörqvist et al. 2012). This is in line with other misophonia research that has demonstrated mixed results with respect to trigger‐specific cognitive deficits. For example, Frank and colleagues (2020) demonstrated that people with misophonia had impairments in alerting attention (achieving and maintaining alertness during a cognitive task) but not orienting attention (directing attention to the source of sensory signals) in the presence of trigger sounds. The cognitive demands of a task, combined with the nature of trigger sound exposure, may play a critical role in determining the extent of trigger‐specific deficits in misophonia. Future research should seek to disentangle how cognitive task type, anticipatory effects, and stimulus duration interact in misophonia.

Some critics have argued that the MisoQuest captures the presence of misophonia but not the severity of symptoms (Rosenthal et al. 2021). We demonstrated that MisoQuest scores correlated significantly with: (1) scores on another measure of misophonia severity (S‐Five); and (2) trigger‐specific reading comprehension deficits, though not with Stroop task performance. These findings may support the use of the MisoQuest as a dimensional measure of misophonia severity. Indeed, Savard and colleagues (2022) demonstrated that English language MisoQuest scores lie on a normally distributed continuum in a general adult sample (*N* = 300), with the cut‐off score identifying only the most severe cases (*n* = 4). Treating misophonia as a dimensional construct allows researchers to capture a wider range of variability, increase statistical power, achieve greater flexibility in statistical modeling, and develop a more nuanced interpretation of individual differences. Despite these advantages, categorical approaches remain relevant to certain research questions (e.g., regarding the prevalence and onset of misophonia) as well as clinical decision‐making. For example, Enzler and colleagues (2021) used the English language MisoQuest's cut‐off score to create comparison groups for the purpose of developing a psychoacoustic test for misophonia. The resulting tool, the Core Discriminant Sounds of Misophonia (CDS_miso_), used trigger sound ratings to separate misophonics and controls with 91% accuracy (Enzler et al. 2021). Researchers and clinicians using the MisoQuest categorically should consider if the high specificity and low sensitivity of the measure aligns with their objectives.

### 4.1 Study Limitations

It is important to interpret these findings in the context of their limitations. First, the same sample was used for both our CFA and EFA. While the CFA was based on the previously identified unidimensional model of the MisoQuest (Siepsiak, Śliwerski, et al. 2020), the use of the same dataset for a subsequent EFA introduces risk of identifying a factor structure that reflects sample‐specific variance rather than a generalizable structure. Independent sample validation would strengthen confidence in the proposed unidimensional structure of the MisoQuest. Next, while the MisoQuest showed strong convergent validity with the S‐Five, there is currently no consensus ‘gold‐standard’ diagnostic tool against which the MisoQuest could be compared. Future studies may opt to include interview‐based assessments (e.g., the Duke Misophonia Interview; Guetta et al. 2022), which might further inform the validity of the MisoQuest.

The online nature of our study presented several challenges. While this approach facilitated recruitment of participants with severe misophonia, it reduced experimental control and likely increased attrition. A total of 46 participants who completed the survey portion of the study were not included in the behavioral analyses, either because they did not complete their assigned task, experienced technical difficulties, or deliberately adjusted their computer volume such that they could not hear experimental sounds. This group had significantly greater misophonia severity than the groups who participated in the behavioural tasks, suggesting our sample may be biased toward those who experienced less distress or managed it more effectively. The resulting smaller sample left our analyses underpowered to detect group differences in cognitive performance.

In contrast, our results were effectively powered when misophonia severity was treated as a dimensional variable. However, there are several task‐specific limitations to consider when interpreting these results. Gum chewing was used as an indicator of misophonia trigger sounds. Although most of our misophonia sample endorsed gum chewing as a trigger, our results may not generalize to other types of trigger sounds. Our null findings on the Stroop task, which diverge from Daniels and colleagues (2020), may reflect methodological differences in stimulus presentation and experimental control. Additionally, we did not screen for color blindness, which could have introduced variability in Stroop task performance. Finally, the reading comprehension task used in this study, while designed by the study team to evaluate story understanding, has not been formally validated.

The current study comprised a sample of 139 participants, including 44 people with misophonia and 95 controls (96 female, 35 male, 8 non‐binary/non‐conforming/agender; 90 White/Caucasian, 10 Black/African Descent, 10 East Asian, 10 South Asian, 3 Middle Eastern/Arab, 3 Latinx/Hispanic, and 13 people from mixed ethnic backgrounds). The misophonic and control groups did not differ significantly in age, ethnicity, education, or reported mental health diagnoses; however, the groups differed in reported household income and gender distribution, with a larger portion of the misophonic sample identifying as female. This aligns with previous research suggesting that misophonia may be more prevalent in females (Dixon et al. 2024). While the overall sample was age and socioeconomically diverse, it was predominantly comprised of white individuals, which may reflect broader demographic trends among English‐speaking populations. Nevertheless, the small sample size limits the generalizability of findings, particularly for underrepresented groups such as Indigenous Peoples and Latinx individuals. Thus, the results represent a step towards validating the English language version of the MisoQuest, but further research with larger and more diverse samples is required to confirm its utility across *all* English‐speaking individuals.

### 4.2 Conclusion

People with misophonia struggle with aversive reactions to everyday sounds. This experience can be psychologically distressing, socially isolating, and since misophonia is not yet recognized in diagnostic manuals, difficult to identify. The present study advanced misophonia assessment by providing a cross‐cultural validation of the MisoQuest in an English‐speaking sample. The results of this study supported the reliability and validity of the English language MisoQuest and demonstrated that scores on this measure predict reading comprehension impairments under trigger sound conditions. These impairments scaled linearly with MisoQuest scores, supporting the utility of the MisoQuest as a dimensional measure of misophonia symptom severity. While we did not replicate previous findings linking misophonia severity to selective attention deficits on the Stroop task, methodological differences may explain this discrepancy. Future research should examine how stimulus presentation and cognitive load influence observed impairments in misophonia. Overall, the present study provides researchers and clinicians with greater confidence in using the MisoQuest to assess misophonia severity in English‐speaking individuals and contributes to growing research on misophonia assessment.

## Conflicts of Interest

The authors declare no conflicts of interest.

## Data Availability

The data that support the findings of this study are openly available in the Open Science Framework at https://osf.io/3pf68/files/osfstorage.
